# Prevalence and determinants of suicidal ideation among South African Health Sciences students at the onset of the COVID-19 pandemic

**DOI:** 10.3389/fpsyt.2025.1492620

**Published:** 2025-03-19

**Authors:** Samhaa Seedat, Muhle Sengwayo, Salma Gani, Lesedi Mashego, Jordan Ochayon, Ashleigh Shepard, Caleb Vergie, Mxolisi Masango, Lerato P. Makuapane, Fezile Wagner, Ryan G. Wagner

**Affiliations:** ^1^ Unit for Undergraduate Medical Education, School of Clinical Medicine, Faculty of Health Sciences, University of the Witwatersrand, Johannesburg, South Africa; ^2^ Analytics and Institutional Research Unit, University of the Witwatersrand, Johannesburg, South Africa; ^3^ Department of Psychology, University of the Witwatersrand, Johannesburg, South Africa; ^4^ Department of Institutional Planning (DIP), University of Pretoria, Pretoria, South Africa; ^5^ South African Medical Research Council/Wits Rural Public Health and Health Transitions Research Unit (Agincourt), School of Public Health, Faculty of Health Sciences, University of the Witwatersrand, Johannesburg, South Africa; ^6^ Barrow Neurological Institute, Phoenix, AZ, United States

**Keywords:** mental health, depression, anxiety, resilience, university, college

## Abstract

**Background:**

Suicidal ideation is an important mental health concern amongst university students who are exposed to multiple stressors. Furthermore, those studying towards degrees in the field of Health Sciences are exposed to additional and unique stressors. This quantitative cross-sectional study aimed to define the prevalence of suicidal ideation and its determinants amongst undergraduate students within the Faculty of Health Sciences at a large, urban South African University.

**Materials and methods:**

An online questionnaire was administered to the 4089 students registered in the Faculty of Health Sciences, with 1211 students submitting responses between March and April 2020. The distribution of this survey coincided with the onset of the national lockdown due to the Covid-19 pandemic. The survey was comprised of validated tools measuring depression (with an included indicator of suicidal ideation), anxiety and resilience. Data analysis was completed using the STATA statistical software (version 14). Both a bivariate analysis and a multivariate logistic regression adjusted for gender were performed.

**Results:**

The total prevalence of suicidal ideation adjusted for gender and race in this sample was found to be 21.8% (CI: 18.6-25.3; p<0.001). Females had significantly higher suicidal ideation than males (24.1% versus 17.8%; p=0.011). In the multivariate model adjusted for gender, having depressive (aOR 10.8; CI: 7.9-14.8; p<0.001) or anxiety (aOR 5.1: CI: 3.8-6.8; P<0.001) symptoms, only sometimes (aOR 2.7: CI 1.8-4.0; p<0.001) or never (aOR 2.7; CI: 1.8-4.0; p<0.001) having anyone to talk to and being of colored race (aOR 2.0; CI: 1.1-3.4; p=0.019) were significantly associated with suicidal ideation, whilst higher resilience (aOR 0.3; CI: 0.3-0.4; p<0.001) was associated with lower levels of suicidal ideation.

**Conclusion:**

The prevalence of suicidal ideation found in this study was higher than that found in the general South African adult population and highlights the need to further investigate and address student mental health, especially students in the field of Health Sciences. Interventions aimed at mitigating the determinants, including depression and anxiety, and promoting the development of protective factors such as resilience and social support may lead to a reduction in suicidal ideation in this population group. Institutional initiatives aimed at improving access to and the quality of mental health services offered to students should be widely advocated for and implemented.

## 1 Introduction

Suicidality encompasses various behaviors related to suicide, including thoughts, plans, attempts, and actual suicides. Suicidal ideation involves thinking about suicide and can be categorized as either active or passive. Passive ideation consists of thoughts about death or suicide, which can represent the initial stage of suicidality, whilst active ideation involves specific plans to carry out the act (1). Whilst suicidal ideation does not always lead to suicidal attempt, ideation can signal significant emotional distress and dysfunction, which is typically associated with underlying mental health issues (2). Further, suicidal ideation is a significant risk factor for future suicide attempts (3).

Suicidal ideation and suicide have a significant and growing global prevalence (4, 5) with suicide ranking as the fourth leading cause of death in individuals aged 15 – 29 years (6). Amongst undergraduate university students, a population largely comprised of young adults, the prevalence of suicidal ideation has been found to be even higher, with at least one in four university students experiencing suicidal ideation (6, 7). Numerous factors for these higher levels amongst students have been suggested and include socio-demographic factors – age, race, socio-economic, religion and sexual orientation (8–10), as well as behavioral factors – sleep disturbances and substance abuse. Suicide attempts amongst family and friends have also been found to be associated with suicidal ideation. Additionally, low resilience, with resilience defined as inter-related qualities that allows one to not only withstand but to thrive in the face of adversity, was found to be significantly associated with suicidal ideation amongst students (11–13). Perhaps most studied, common mental disorders, including anxiety and depression, have persistently be shown to be significantly associated with suicidal ideation, with depression being one of the strongest predictors (8, 14). Worldwide studies have shown markedly high levels of these common mental disorders in university students (15).

Entry into the university environment often coincides with a time of intense changes in responsibilities and demands, requiring rapid adaptation. Coupled with this is the potential emergence of other mental health conditions (16). These new stressors combined with often evolving or even lack of support systems (17) can lead to increased levels of anxiety, depression and substance abuse. These stressors can be even more pronounced amongst Health Science students, particularly those who are patient-facing, who experience heightened stressors including providing patient care, dealing with suffering and death in an often high stress environment. These stressors were likely exacerbated during the COVID-19 pandemic due to the increased severity of illness among their patient population as well as the likely impact that the pandemic had on both their personal and professional lives (18–20). Previous research has found that quarantine and isolation significantly contribute to the poor mental health of individuals (21). Since most Health Science students are working in the clinical space during their degree, proximal triggers from working in hospitals and clinics may be more pronounced during disease outbreaks and pandemics (22).

In addition to understanding the socio-demographic, behavioral, psychological and temporal determinants of suicidal ideation, understanding the contextual determinants are also important. Studies have shown that low- and middle-income countries, including South Africa, carry the highest burden of suicide risk for students in the field of Health Sciences, with higher levels of suicidal ideation amongst medical students found in low-income countries compared to high-income countries (8, 23). In the South African academic setting, students may be faced with stressors such as financial adversity, exposure to violence, or coming from challenging familial backgrounds that can not only impact academic performance but have lasting effects on their wellbeing (24–26). The prevalence of suicidal ideation among university students in South Africa was found to be much higher than the general adult population, with a 40.9% prevalence among students compared to a 9.1% prevalence among adult South Africans (25). These results were also higher than that reported in international literature (27). Students in the field of Health Sciences may be further burdened, with one study finding 32.3% of medical students reporting suicidal ideation (8). Yet research on suicidal ideation amongst South African Health Sciences students remains limited, but is anticipated to be substantial given previous studies that found 25% of South African medical students met the clinical criteria for Major Depressive Disorder and 20.5% met the criteria for a clinical diagnosis of Generalized Anxiety Disorder, with the latter significantly associated with a greater likelihood of suicidal ideation (aOR 4.53; p<0.001) (28, 29). It should be acknowledged that these studies were undertaken prior to the COVID-19 pandemic and as noted above, we would anticipate the pandemic to exacerbate this substantial burden.

Given the limited research on suicidal ideation amongst Health Science students in South Africa, particularly during the COVID-19 pandemic and the fact that suicidal ideation is an important indicator of predicting future suicide attempts (25, 27), we aim to quantify the burden and identify the determinants of suicidal ideation in this population. The findings can help to inform the development of targeted mental health interventions. Studies have shown increased levels of burnout and suicide among both university students and residents globally (30, 31). Understanding the prevalence of suicidal ideation during these formative years of training may allow for the development of programs that provide support and foster resilience, thereby improving students’ lifelong wellbeing.

## 2 Materials and methods

### 2.1 Study context

This study took place at a large, research intensive, urban South African university with over 40,000 students. The student body is diverse and represents all South African race groups. Sixty percent of students are enrolled in undergraduate programs and registered in one of five Faculties - the Faculty of Commerce, Law and Management; the Faculty of Engineering and the Built Environment; the Faculty of Health Sciences; the Faculty of Humanities; and the Faculty of Science. In 2020, 17% of students were registered in the Faculty of Health Sciences (32). The Faculty of Health Sciences offers a wide variety of undergraduate degree programs including biokinetics, biomedical sciences, clinical medical practice, dentistry, health systems sciences, medicine and surgery, nursing, occupational therapy, oral hygiene, pharmacy and physiotherapy. The duration of undergraduate degrees ranges between 3 and 6 years of fulltime study and the majority involve dedicated time in the clinical environment with students receiving training at government-run, public hospitals and clinics (33). The current study took place during the Alert Level 5 lockdown in South Africa during the Covid19 pandemic in 2020 (34). As a result, all higher education contact activities were halted (35). Despite the lockdown, students registered in the field of Health Sciences who were in their clinical years continued their clinical training under strict safety measures.

### 2.2 Study sample

The study sample comprised of students who were 18 years or older, registered full time in the Faculty of Health Sciences during the year 2020, enrolled for an undergraduate program and had a full biographic record in the university’s data warehouse. Students who did not meet the inclusion criteria were excluded from the analytical sample.

### 2.3 Study procedures

Following approval from the University’s ethics committee as well as the University registrar, a list of students meeting the inclusion criteria was requested from the University. Data collection took place between March and April 2020 and students who were eligible to participate received an email inviting them to participate in the study. Included in the email was information about the study and a link to the online, self-administered survey, delivered on the REDCap platform (36). Students who accepted to participate via an online consent process were then directed to the survey. Students who did not participate within the first week received an automated follow-up reminder email one week later.

### 2.4 Instruments

Several tools were included in the online survey and examined depressive symptomology, suicidal ideation, anxiety and resilience.

#### 2.4.1 Socio-demographic variables

The socio-demographic characteristics included: age (categorized as <20, 21, 22, 23, 24 or >24 years), year of academic study (YOS 1-6), high school location (rural or urban), home location (rural village or farm, informal settlement or township, or city or suburb), program type (General Academic 1st Bachelor’s Degree or Professional 1st Bachelor’s Degree), residence status (on or off campus), disability status (yes or no), race (African, White, Indian, Colored, Chinese), high school quintile [a system used to classify schools in the South African context from poorest (1) to wealthiest (5) and Private/International/Other (6)], first generation status indicating whether students were first in their family to attend university (first generation or non-first generation).

#### 2.4.2 Depressive symptoms

Depressive symptoms were measured with the Patient Health Questionnaire 9 (PHQ-9). The PHQ-9 is a 9-item depression screening questionnaire measuring depressive symptoms as defined by the DSM V (2). Participants are required to rate each of the 9 items or symptoms based on: “Over the last 2 weeks, how often have you been bothered by any of the following problems.” The anchors used to rate each item range from ‘0-Not at all’ to ‘3-Nearly every day’. Aggregated PHQ-9 scores of 0–4 indicate minimal depressive symptoms; 5–9 indicate mild depressive symptoms; 10–14 indicate moderate depressive symptoms; 15–19 indicate moderate-severe depressive symptoms and 20–27 indicate severe depressive symptoms. Participants’ responses were totaled and assigned to one of these five categories.

Depression was present if scores were equal to or above the threshold of 10, indicative of the presence of moderate depressive symptoms (37). These categories have previously been employed in research with South African university students (38). The PHQ-9 has been validated for use in the South African context in both a community setting (39) and the primary healthcare setting (40). It has also been validated for use in university students in the Bangladeshi (41) and Nigerian (42) contexts.

#### 2.4.3 Suicidal ideation

The ninth item of the PHQ-9 tool was used to assess for suicidal ideation and asks, “How often have you been bothered by thoughts that you would be better off dead or hurting yourself in some way?”. Like the other questions in the PHQ-9, the answer for this question was graded on a four-point Likert scale, ranging from ‘0-Not at all’ to ‘3-Nearly every day’. Participants scoring at least 1 on this item were coded for suicidal ideation. An additional question was posed to those exhibiting suicidal ideation: “Do you have a plan on how you would take your life?”. Those that responded ‘yes’ to the additional question were defined as having active suicidal ideation and those answering ‘no’ were defined as having passive suicidal ideation. These group of respondents were flagged for immediate intervention from the faculty and university counselling services.

#### 2.4.4 Anxiety symptoms

The Generalized Anxiety Disorder (GAD-7) scale measures symptoms of anxiety as outlined in the DSM-V (2). Respondents are asked to reflect on how often, during the past two weeks, they have been bothered by each symptom. Response format ranges from ‘0-not at all’ to ‘3-nearly every day’ (43). Like the PHQ-9, responses from the GAD-7 were summed and assigned to one of four GAD-7 categories with severity scores as follows: a score of 0-4 indicating minimal anxiety, 5-9 indicating mild anxiety, 10-14 indicating moderate anxiety, and 15-21 indicating severe anxiety. These categories have previously been employed in research conducted with South African undergraduate students and validated in a study of postgraduate Chinese medical students (44, 45).

#### 2.4.5 Resilience

A modified Brief Resilience Scale (BRS) measures the unitary construct of resilience and seeks to assess an individual’s ability to recover from stressful situations (46). This 6-item scale has response options ranging from ‘1-Strongly disagree’ to ‘5-Strongly agree’, with higher scores indicating higher levels of resilience (46). According to Smith et al. internal consistency reliability amongst four samples produced Cronbach alpha values between 0.80 and 0.91. In addition, this score also favorably correlates with other measures of optimism and resilience (46).

### 2.5 Data analysis

Both descriptive and multivariate statistical analyses was undertaken using STATA (version 14). Descriptive data are presented as means and standard deviations (SD) for normally distributed data and medians and interquartile ranges for non-normally distributed data. Prevalence figures are reported by gender with 95% confidence intervals. Weighted prevalence figures, accounting for gender and race differences between respondents and the underlying student population are also presented. Chi-squared tests were undertaken to identify associations between the possible determinants and suicidal ideation; bivariate and multivariate logistical regressions were used to explore determinants with a p-value <0.05 considered statistically significant.

### 2.6 Ethical considerations

Approval for this study was granted by the Human Research Ethics Committee of the University of the Witwatersrand (Non-medical - protocol number: H18/11/44 and Medical - protocol number: M2010121) and the University Registrar. All study information was included in the body of the invitation email. All participants gave their informed consent prior to completing the questionnaire. Additionally, participants received contact information for psychological counselling providers that they could contact if necessary and if participants were found to be experiencing suicidal ideation or severe depressive symptomology, they were offered further contact with student support systems.

## 3 Results

A total of 1211 responses were received from a possible 4089 Health Science students, a response rate of 29.6%.

### 3.1 Demographics

Respondents were mostly female (73.6%), with the largest race group being African (48.8%). The sample included more students per year of study (YOS) in YOS 1 to 4 than those in YOS 5 and 6 (15.0%-25.9% each for YOS 1-4; 7.6% - 7.9% each YOS 5-6). Most students attended high school in an urban area (73.8%) and a small majority (51.3%) reported that their home was in a city or suburb. Most respondents (71.0%) were not residing in university residences. Those with disabilities made up 3.1% of the sample. In terms of first-generation status, 24.6% indicated that they were first in their family to attend university (**Table 1**). The majority of students were in the Professional Bachelor’s degree program (78.0%).

**Table 1:** Demographic characteristics of participants by gender.

Socio-demographic Variable	Female n (%) 892 (73.6%)	Male n (%) 319 (26.3%)	Total n (%) 1211 (100%)
Age (in years)			
< 20	216 (79.4%)	56 (20.1%)	272 (22.5%)
20	157 (77.7%)	45 (22.3%)	202 (16.7%)
21	143 (81.2%)	33 (18.8%)	176 (14.5%)
22	120 (69.0%)	54 (31.0%)	174 (14.4%)
23	85 (63.9%)	48 (36.1%)	133 (10.1%)
24	75 (70.8%)	31 (29.3%)	106 (8.8%)
> 24	96 (64.9%)	52 (35.1%)	148 (12.2%)
Year of Study			
1	244 (78.0%)	69 (22.0%)	313 (25.9%)
2	146 (76.4%)	45 (22.6%)	191 (15.8%)
3	228 (76.7%)	69 (23.2%)	297 (24.5%)
4	122 (67.4%)	59 (32.6%)	181 (15.0%)
5	60 (62.5%)	36 (37.5%)	96 (7.9%)
6	62 (67.4%)	30 (32.6%)	92 (7.6%)
Unknown	30 (73.2%)	11 (26.8%)	41 (3.4%)
High School Location			
Rural	104 (65.8%)	54 (34.2%)	158 (13.1%)
Urban	679 (76.0%)	215 (24.1%)	894 (73.8%)
Unknown	16 (72.7%)	6 (27.3%)	22 (1.8%)
Home Location			
Rural Village or Farm	88 (68.2%)	41 (31.2%)	129 (10.1%)
Informal Settlement	2 (66.7%)	1 (33.3%)	3 (0.3%)
Township	119 (77.3%)	35 (22.7%)	154 (12.7%)
City/Suburb	478 (77.0%)	143 (23.0%)	621 (51.3%)
Program Type			
General 1^st^ bachelor’s degree	205 (77.1%)	61 (22.9%)	266 (22.0%)
Professional 1st bachelor’s degree	687 (72.7%)	258 (27.3%)	945 (78.0%)
In University Residence			
No	639 (74.3%)	221 (25.7%)	860 (71.0%)
Yes	253 (72.1%)	98 (27.9%)	351 (29.0%)
Disability			
Yes	30 (79.0%)	8 (21.1%)	38 (3.1%)
No	862 (73.5%)	311 (26.5%)	1173 (96.9%)
Race			
African	442 (74.8%)	149 (25.2%)	591 (48.8%)
White	256 (73.4%)	93 (26.7%)	349 (28.8%)
Indian	141 (67.8%)	67 (32.2%)	208 (17.2%)
Colored	44 (81.5%)	10 (18.5%)	54 (4.5%)
Chinese	9 (100.0%)	0 (0.0%)	9 (0.7%)
High School Quintiles			
1	19 (65.5%)	10 (34.5%)	29 (2.4%)
2	41 (61.2%)	26 (38.8%)	67 (5.5%)
3	54 (65.9%)	28 (34.2%)	82 (6.8%)
4	52 (70.3%)	22 (29.7%)	74 (6.1%)
5	346 (77.1%)	103 (22.9%)	449 (37.1%)
Private/International/Other	380 (74.5%)	130 (25.5%)	510 (42.1%)
First Generation Status			
First generation	231 (77.5%)	67 (22.5%)	298 (24.6%)
Non-first generation	456 (74.9%)	153 (25.1%)	609 (50.3%)
Unknown	205 (67.4%)	99 (32.6%)	304 (25.1%)

### 3.2 Prevalence of suicidal ideation

The overall weighted prevalence of suicidal ideation amongst undergraduate Health Sciences students was 22.0% (CI: 19.8-24.5). Females reported a significantly higher level of suicidal ideation (24.1%; CI: 21.3-27.2) compared to their male counterparts (17.8%; CI: 14.4-21.9; p=0.011). In addition, the weighted prevalence of suicidal ideation was significantly higher amongst Colored respondents (35.9%; CI: 24.7-51.2) compared to White respondents, who had the lowest levels of suicidal ideation (19.1%; CI: 15.0-24.0; p=0.036) (**Table 2**). Differentiation by type of ideation showed that active ideation (20.7%; CI: 16.3-26.0) was less prevalent than passive ideation (79.2%; CI: 74.0-83.7).

**Table 2:** Bivariate analysis – demographic factors by suicidal ideation.

Socio-demographic Variable	Total (n)	Suicidal ideation n (%)	95% CI	*p-*value
Gender				
Female	892	214 (24.1%)	21.3 -27.2	0.011
Male	319	56 (17.8%)	14.4 -21.9	
Race				
African	591	135 (22.8%)	19.5 -26.4	0.036
White	350	68 (19.1%)	15.0 -4.0	
Indian	208	46 (22.1%)	16.7 -28.7	
Colored	54	20 (35.9%)	24.7-51.2	
Chinese	9	2 (22.2%)	2.8-60.0	
High School Quintiles				
1	29	5 (17.2%)	5.9 -35.8	0.012
2	67	17 (25.3%)	15.5 -37.5	
3	82	30 (36.6%)	26.2 -48.0	
4	74	12 (16.2%)	8.7 - 16.6	
5	449	106 (23.6%)	19.8 -27.8	
International/private unknown	510	100 (19.6%)	16.3 -23.3	
Disability Status				
With	38	18 (47.3%)	31.0 -64.2	<0.001
Without	1174	253 (21.6%)	19.2 -25.0	
Presence of Depressive Symptoms				
No	903	97 (10.7%)	8.8 -13.0	<0.001
Yes	309	174 (56.3%)	50.6 -61.9	
Presence of Anxiety Symptoms				
No	765	89 (11.6%)	9.5 -14.1	<0.001
Yes	447	182 (40.7%)	36.1 -45.4	
Resilience				
Low	387	153 (39.5%)	34.6 -44.6	<0.001
Normal	717	108 (15.1%)	12.5 -17.9	
High	108	10 (9.3%)	4.54 -16.4	
Someone To Talk To				
Often	334	40 (12.0%)	8.7 - 16.0	<0.001
Sometimes	591	158 (26.7%)	23.2 - 30.5	
Never	287	73 (25.4%)	20.5 - 30.9	
First Generation Status				
First Generation Student	298	74 (24.8%)	20.0 - 30.1	0.068
Non-first generation	609	119 (19.5%)	16.5 - 22.9	
Unknown	304	77 (25.3%)	20.5 -30.6	
In University Residence				
No	860	180 (20.9)	18.3 – 23.8	0.074
Yes	351	90 (25.6)	21.2 – 30.5	

### 3.3 Depression and anxiety

Results found that 309 (28.0%) respondents reported symptoms of depression. Of those who reported symptoms of depression, a significant proportion of student respondents (56.3%) also reported suicidal ideation (CI: 50.6-61.9; p<0.001). Similarly, of the 447 student respondents (36.9%) who reported symptoms of anxiety, 40.7% reported suicidal ideation (CI: 36.1-45.4; p<0.001) (**Table 2**).

### 3.4 Resilience

Individuals with lower resilience scores (39.5%; CI: 34.6-44.6; p<0.001) had significantly higher levels of suicidal ideation than those with higher resilience scores (9.26%; CI: 4.5-16.4; p<0.001) (**Table 2**).

### 3.5 Social support

Student respondents were asked to classify whether they had someone to talk to, which was categorized into *often*, *sometimes* or *never*. Students who reported having someone to talk to *sometimes* (26.7%; CI: 23.2-30.5) or *never* (25.4%; CI: 20.5-30.9) had a significantly higher prevalence of suicidal ideation compared to those who had someone to talk to *often* (12.0%; CI: 8.7-16.0; p<0.001) (**Table 2**).

### 3.6 Multivariate logistic regression, adjusted for gender

After adjusting for gender, the logistic regression found that having depressive symptoms (aOR 10.8; CI: 7.9-14.8; p<0.001), generalized anxiety symptoms (aOR 5.1; CI: 3.8–6.8; p<0.001), being a colored student (aOR 2.0; CI: 1.1–3.4; p=0.019), only *sometimes* having someone to talk to (aOR 2.7; CI 1.8-4.0; p<0.001) or *never* anyone to talk to (aOR 2.7; CI: 1.8-4.1; p<0.001) were significantly associated with suicidal ideation, whilst higher resilience (aOR 0.3; CI: 0.3-0.4; p<0.001) was significantly associated with lower levels of suicidal ideation (**Table 3**).

**Table 3:** Multivariate logistic correlates to suicidal ideation amongst respondents (adjusted for gender).

Sociodemographic Variables	Odds ratio (95% CI)	*p*-value
Race (ref: African)		
Chinese	0.9 (0.1-7.3)	0.913
Colored	2.0 (1.1-3.4)	0.019
Indian	1.0 (0.7-1.4)	0.997
White	0.8 (0.6-1.2)	0.246
High School Quintiles (ref: Quintile 1)		
2	2.1 (0.7-6.3)	0.211
3	3.1 (1.1-9.2)	0.041
4	1.1 (0.3-3.4)	0.928
5	1.6 (0.6-4.6)	0.338
(International/Private/Other)	1.7 (0.5- 3.8)	0.552
Presence of Depressive Symptoms (ref: No)		
Yes	10.9 (7.9-14.8)	<0.001
Presence of Anxiety Symptoms (ref: No)		
Yes	5.1 (3.8-6.8)	<0.001
Resilience (ref: No)		
Yes	0.3 (0.2-0.4)	<0.001
Someone to talk to (ref: Often)		
Sometimes	2.7 (1.8-4.0)	<0.001
Never	2.7 (1.8-4.1)	<0.001
First Generation Status (ref: Non-first generation)		
First generation	0.8 (0.6-1.1)	0.184
In University Residence (ref: No)		
Yes	1.3 (1.0-1.8)	0.067

## 4 Discussion

### 4.1 Suicidal ideation

The prevalence of suicidal ideation among university students, and particularly Health Science students, has been shown to be substantial and a growing concern worldwide (47). Previous work from South Africa, where students are exposed to various structural and environmental stressors have shown high levels of depression and anxiety, with the COVID-19 pandemic thought to exacerbate these (48). That said, these factors in this context and student population have not been rigorously explored. As such, we sought to determine the prevalence of suicidal ideation and its determinants among Health Science students at a large South African University.

We found the adjusted prevalence of suicidal ideation in our Health Science student population to be 22%. This figure is comparable to the estimated lifetime prevalence of suicidal ideation among students across 36 institutions in both high- and low-income countries (49). Notably, the prevalence of suicidal ideation that we found was lower than that found by other South African researchers who reported a prevalence of suicidal ideation of 32.3% among medical students using questions from the standardized Paykel instrument (28). This difference may be due to the tool used to estimate suicidal ideation or the fact that the earlier study focused solely on medical students and not all Health Science students. In the university setting, increased academic demands coupled with the pressure to excel and self-imposed expectations can leave many at risk (50). Students who are patient-facing may experience similar pressures as healthcare workers such as physicians who are at high-risk for suicide (51). Occupational factors in the health profession are implicated in the increase of distress and psychopathology leading to suicidal thoughts. These include increased workload coupled with long hours and the intensity of stress conditions with access to drugs to facilitate suicidal acts are implicated in triggering suicidal thoughts (51). Burnout is a common feature among medical students, which results in emotional exhaustion and depersonalization as a result of an intensified workload in the healthcare setting (52). This not only impacts quality of care but often results in suicidal tendencies (52).

The unique South African context may serve to exacerbate these stressors, with the university space serving as a microcosm of the inequities that persist; a space in which prevailing stressors such as socioeconomic inequality and trauma are concentrated and brought to the fore (53). In addition to the distal stressors that exist in South Africa, university students are further exposed to proximal stressors within their university environment. This is evidenced in work by Bantjes et al., who reported that South African students were at a greater risk of suicidal ideation than the general South African population (54). Whilst not found to be significant in the multivariate model, finding a higher prevalence of suicidal ideation amongst first generation University students may be indicative of the unique South African context.

### 4.2 Socio-demographic determinants

We found that female students reported higher levels of suicidal ideation compared to their male colleagues. This finding is in keeping with work from Bantjies et al. (RR 1.9, 95% CI: 1.3 - 2.7) in their analysis of the South African National Student Mental Health Survey which was conducted across 17 of the 26 public universities in the country (45). These findings, often reflected globally, are likely a consequence of a confluence of factors including psychosocial stressors (55, 56) higher rates of common mental disorders (57, 58), and increased risk for sexual assault and harassment (59). Our analysis shows that females continued to experience higher levels of suicidal ideation during the COVID-19 pandemic, but being female was not the only associated demographic factor.

Although there was an unequal number of respondents across the different racial groups, a proportion of individuals reporting suicidal ideation belonged to historically disadvantaged racial groups, which in the South African context includes Colored and Black African respondents. This is supported by a study conducted in the United States of America by Oh et al. (60), which analyzed suicidal ideation in minority (Asian, Latino and Black) groups and demonstrated that these groups experienced increased levels of discrimination; directly contributing to their increased prevalence of suicidal ideation. Cover (61) notes that discrimination and marginalization - which in the South African context may be linked to the socioeconomic disparities created by *Apartheid* and which have largely remained unaltered since the advent of democracy - are associated with an increased risk of suicidal behavior.

### 4.3 Depression and anxiety

We found levels of depression and anxiety symptoms similar to previous studies conducted with University of Cape Town medical student- 36.4% and 45.9%, respectively (28). The slightly higher rates reported in that study, compared to ours, may in part be explained by two factors - the academic disruptions following the Fees Must Fall protests (a student led protest against tertiary education fees) and the death by suicide of Professor Bongani Mayosi, Dean of the Faculty of Health Sciences at the University of Cape Town shortly before that research was conducted (53).

In a systematic review of psychological autopsies conducted by Cavanagh et al., mental disorders, of which depression was the most common, were found to be strongly associated with suicide. Approximately 90% of suicide victims had depressive symptoms (62). Our analysis shows that depression is significantly associated with suicide ideation. A study by Santos et al., conducted in the United States of America, showed that 2% of study respondents had suicidal ideation during their academic career and of those with suicidal ideation, 17% reported depressive symptoms (27). A study amongst Nigerian university students found a 22.5% prevalence of depression and 21.6% prevalence of suicide ideation with a statistically significant (p<0.001) association between these two (63). Importantly, both of these studies were conducted amongst university students in general and not specifically focused on Health Science students.

### 4.4 COVID-19

Fears around infection and death, economic uncertainty and social isolation during the pandemic likely contribute to the development of psychiatric disorders such as anxiety and depression, which can lead to the development of suicidal ideation (64–66). Due to the design of our study, we are not able to directly determine the effect of the COVID-19 pandemic on levels of depression, anxiety and suicidal ideation, but are able to conclusively state that depression and anxiety symptoms are associated with suicidal ideation- and all three remained prevalent during the COVID-19 pandemic. Several studies have previously reported elevated levels of anxiety and depression among university students during the COVID-19 pandemic. A meta-analysis of 27 global studies measuring University student mental health during the pandemic reported an overall prevalence of depression and anxiety to be 39% (95% CI: 27-51%) and 36% (95% CI: 26-46%), respectively, with higher levels reported in a low-income country and found to be generally in line with our findings, with our study showing a lower prevalence of depression symptoms (28.0%) (20, 67). Some of the observed differences may be a consequence of the different tools and definitions used to determine depression and anxiety – a topic further discussed in the *Strengths and Limitations section* towards the end of this manuscript.

Among medical students specifically, fear of COVID-19 infection, poor health literacy, dissatisfaction with online learning and associated difficulties following online learning were all found to negatively impact the mental health of medical students (68). While at least one study found increased levels of suicidal ideation during the initial COVID-19 outbreak, there has been no definitive link found between increased suicidal ideation and the pandemic in the general population (69). It is suggested that targeting depression and anxiety during times of severe distress can aid in the reduction of suicidal ideation and suicide risk (65).

### 4.5 Resilience

We found resilience to be a protective factor against suicidal ideation. Resilience is the modifying factor through which other influencing factors of suicidal ideation should be viewed. An individual with strong resilience is significantly less likely to be suicidal, even in the face of substantial stressors (13). A study of undergraduate medical students conducted at the University of the Free State in South Africa found that academic stress was associated with low resilience (70). In addition, black, pre-clinical, first-generation and female students scored lower for resilience, suggesting that targeted interventions aimed at improving resilience may have the dual effect of increasing resilience and decreasing suicidal ideation amongst the most vulnerable groups of students.

### 4.6 Strengths & limitations

This study had several notable strengths including a relatively high response rate when compared to similar online studies amongst university students. Furthermore, standard tools, including the PHQ-9, GAD-7 and Brief Resilience Scale were used to assess depression symptoms, anxiety symptoms and resilience, respectively. Finally, the respondents of this study represent a unique and understudied population- Health Science students at a South African University during the time of the COVID-19 pandemic.

In addition to the strengths, several weaknesses should also be noted. The outcome of interest – suicidal ideation- was firstly determined using a single item construct in the PHQ-9, which has been shown in at least one study to have limited utility in certain sub-groups and clinical settings resulting in high levels of false-positive responses. This previous study goes on to recommend that a validated suicide risk inventory, such as the Columbia Suicide Severity Rating Scale, should be used in conjunction with the PHQ-9 (71). Machine learning algorithms have suggested that using the 9^th^ item of the PHQ in a primary health care setting is reliably accurate and, given its previous use, can allow for useful comparison (72). That said, we acknowledge this as a limitation and further work should seek to the validate the specificity of the tool in this population. Similarly, the Brief Resilience Scale (46) has limited psychometric properties published in South African and student populations; hence a more robust and psychometrically valid resilience tool may be used in future studies to ensure reliability and conceptual consistency in measuring this construct in South African student populations.

The unique demographics in our sample restrict our ability to generalize the findings to other population groups. A greater proportion of females than males (when compared to the underlying population) participated in this study. We overcame this limitation by adjusting our overall prevalence estimates by gender and weighted means across the study population. Another limitation was the potential presence of bias. Social bias may be present as suicidal ideation and its determinants may be negatively perceived and thus underreported with cultural nuances influencing this bias. Recall bias may be present as the questionnaire requires recall of events and feelings over a two-week period which may not be accurate.

Finally, potential mental distress and suicidal ideation precipitants are inherently fluctuating in nature; students may face increased stressors around assessment periods or during certain clinical rotations in which their exposure death and trauma is heightened. As a result of the cross-sectional nature of this study we are unable to comment on possible variations in data if they were to be collected at differing points through the academic year or as students progress through each year of their studies. Further studies should seek to more fully assess differing factors and their effect on the mental health of Health Science students during different time points of the academic year. Lastly, due to the cross-sectional nature of this study, there was no manipulation of the independent variable, and an absence of temporal precedence precludes causality between the variables investigated.

### 4.7 Future directions

The implications of these mental health challenges are far-reaching. The personal, social and academic well-being of Health Science students is directly impacted by their mental health during their training years of training. However, the consequences extend beyond their immediate experience and could have long-term effects on the quality of care they provide as future healthcare professionals (73). Students in their clinical years are front-line workers who are responsible for the provision of healthcare services under supervision and as such those struggling with their mental health may not be able to provide services of the requisite standard, which could have wider repercussions for the healthcare system. Long-term implications of student mental health must be considered as these individuals will constitute the healthcare workforce in future years, this research can serve as a catalyst for providing mental health services for healthcare trainees.

To reduce the high prevalence of suicidal ideation in the student population, it is crucial to identify and address both the determinants and protective factors. This knowledge should be shared with both students and academic and healthcare professionals responsible for student wellness. This awareness should inform that design of interventions aimed at improving mental health and mitigating suicidal ideation.

Depression and anxiety, found to be significantly associated with suicidal ideation in this study, can be addressed by improving access to mental health support services. Students should be trained to recognize the early signs of mental distress, particularly when faced with stressors in clinical environments. Regular self-screening for mental health concerns should be incorporated into students’ routines, along with periodic formal mental health assessments and timely referrals to professional services when needed. Beginning from the first year of study, targeted workshops and mentorship programs focused on managing the unique stressors of Health Sciences training could help prevent the development of serious mental health issues and reduce stigma around seeking help. Additionally, resilience and social support, the identified protective factors, should be fostered through continuous academic and social support, alongside the creation of a university culture that emphasizes motivation, pride, and community. Initiatives like mentorship programs and team-building workshops can play a vital role in cultivating this supportive environment. Finally, when implementing these strategies, it is important that those providing assistance are sensitive to the demographic factors associated with suicidal ideation identified in our study, ensuring that interventions are both inclusive and tailored to the needs of the most vulnerable student groups.

## 5 Conclusions

We have shown that suicidal ideation amongst Health Sciences students in South Africa during the COVID-19 pandemic was common and, together with evidence from previously published research, suggests that it is likely to persist. Future research is needed to determine the temporality of certain determinants including symptoms of depression and anxiety. Interventions that increase resilience should be directed to towards minority groups who were found to have the largest prevalence of suicidal ideation. Strengthening resilience and providing comprehensive mental health care support for Health Sciences trainees may mitigate the burden of suicidal ideation in this population, thereby improving their well-being.

## Data Availability

The datasets presented in this article are not readily available as additional ethics may be required to allow access to dataset. Requests to access the datasets should be directed to RGW, ryan.wagner@wits.ac.za.
